# Pathophysiology and Treatment of Lipid Abnormalities in Cerebrotendinous Xanthomatosis: An Integrative Review

**DOI:** 10.3390/brainsci13070979

**Published:** 2023-06-22

**Authors:** Rodrigo Mariano Ribeiro, Sophia Costa Vasconcelos, Pedro Lucas Grangeiro de Sá Barreto Lima, Emanuel Ferreira Coelho, Anna Melissa Noronha Oliveira, Emanuel de Assis Bertulino Martins Gomes, Luciano de Albuquerque Mota, Lucas Soares Radtke, Matheus dos Santos Carvalho, David Augusto Batista Sá Araújo, Maria Suelly Nogueira Pinheiro, Vitor Carneiro de Vasconcelos Gama, Renan Magalhães Montenegro Júnior, Pedro Braga Neto, Paulo Ribeiro Nóbrega

**Affiliations:** 1 Faculty of Medicine, Federal University of Ceara, Fortaleza 60430-372, Brazil; rodrigo.marianoribeiro@hotmail.com (R.M.R.); sophia.costava@gmail.com (S.C.V.); pedro.lucas@alu.ufc.br (P.L.G.d.S.B.L.); emanuelcoelho@alu.ufc.br (E.F.C.); melissanoronha@alu.ufc.br (A.M.N.O.); emanueldeassis@alu.ufc.br (E.d.A.B.M.G.); lucianomota@alu.ufc.br (L.d.A.M.); lucasradtke2031@alu.ufc.br (L.S.R.); matheus.oak@alu.ufc.br (M.d.S.C.); daviaugusto1889@gmail.com (D.A.B.S.A.); msuellynogueirap@gmail.com (M.S.N.P.); vitorgama907@gmail.com (V.C.d.V.G.); 2 Walter Cantídio University Hospital, Federal University of Ceara/Ebserh, Fortaleza 60430-372, Brazil; renanjr@ufc.br; 3 Division of Neurology, Department of Clinical Medicine, Federal University of Ceara, Fortaleza 60430-372, Brazil; paulo_r_med@yahoo.com.br

**Keywords:** cerebrotendinous xanthomatosis, cholestanol, cholesterol, lipid, chenodeoxycholic acid

## Abstract

Cerebrotendinous xanthomatosis (CTX) is an autosomal recessive disorder caused by pathogenic variants in *CYP27A1*, leading to a deficiency in sterol 27-hydroxylase. This defect results in the accumulation of cholestanol and bile alcohols in various tissues, including the brain, tendons and peripheral nerves. We conducted this review to evaluate lipid profile abnormalities in patients with CTX. A search was conducted in PubMed, Embase and the Virtual Health Library in January 2023 to evaluate studies reporting the lipid profiles of CTX patients, including the levels of cholestanol, cholesterol and other lipids. Elevated levels of cholestanol were consistently observed. Most patients presented normal or low serum cholesterol levels. A decrease in chenodeoxycholic acid (CDCA) leads to increased synthesis of cholesterol metabolites, such as bile alcohols 23S-pentol and 25-tetrol 3-glucuronide, which may serve as surrogate follow-up markers in patients with CTX. Lipid abnormalities in CTX have clinical implications. Cholestanol deposition in tissues contributes to clinical manifestations, including neurological symptoms and tendon xanthomas. Dyslipidemia and abnormal cholesterol metabolism may also contribute to the increased risk of atherosclerosis and cardiovascular complications observed in some CTX patients.

## 1. Introduction

Cerebrotendinous xanthomatosis (CTX) is a rare lipid storage disorder related to bile acid synthesis pathways. This autosomal recessive disease is caused by pathogenic variants in *CYP27A1*, which codes sterol 27-hydroxylase, an enzyme of the cytochrome P450 oxidase system with an important role in cholesterol metabolism and bile acid synthesis. Reduced activity of this enzyme leads to increased lipid content formation and accumulation in multiple tissues, which are mainly the brain, eye lenses and tendons [1,2].

Despite being considered rare, CTX has recently been pointed out as considerably underdiagnosed, which might be related to the high heterogeneity of clinical presentation with a wide range of symptoms, severity and age of onset [3,4]. Typical manifestations include tendon xanthomas, osteoporosis, coronary heart disease and progressive neuropsychiatric symptoms. The neurologic disturbances comprise peripheral neuropathy, pyramidal signs (such as spastic paraparesis), cerebellar ataxia, cognitive impairment and movement disorders. In childhood and adolescence, the primary findings are bilateral juvenile cataracts, chronic diarrhea and intellectual disability [2,3,5]. It should be noted that one of the earliest manifestations of CTX is neonatal cholestatic jaundice, a condition that, although self-limited and uncomplicated in most cases, occasionally progresses to a severe form with the requirement for liver transplantation and high lethality [2,6].

The presence of two of the four main characteristics (cataract, diarrhea, progressive neurological involvement and tendon xanthomas) in young individuals incites a more thorough biochemical study for CTX [7]. In this context, the main plasmatic marker is the increase in cholestanol serum levels [4], although it is expected that other substances are also altered, such as low or normal cholesterol serum levels and increased plasmatic and urinary levels for bile alcohols [8,9]. Radiologic analysis can also strengthen the diagnostic suspicion of CTX. The most typical findings are symmetrical abnormalities in the dentate nucleus, as shown by T2W and FLAIR brain MRI [10]. About 84% of CTX patients present an abnormal neuroimage, although these abnormalities may manifest late in the disease’s course [5]. Some diagnostic criteria have been proposed, among which the Mignarri suspicion index must be highlighted [11]. The presence of biallelic pathogenic *CYP27A1* variants and elevated cholestanol, performed in a suggestive clinical context, establishes the diagnosis of CTX [12].

CTX treatment is carried out with chenodeoxycholic acid (CDCA), which inhibits the accumulation of cholestanol by reducing bile acid production [7]. It mainly aims to stabilize the progression of neurological manifestations but may also lead to symptom improvement in some patients [13]. The establishment of an early treatment seems to influence the therapeutic response positively [14]. On the other hand, some individuals with CTX continue to have their neurological function deteriorate even after the beginning of treatment with CDCA, especially when it is started late in the disease’s course [15].

This study aims to describe the biochemical characteristics of the lipid profile in CTX, elucidating the aspects of cholesterol metabolism, pathophysiology, molecular diagnosis and biochemical changes after CTX treatment.

## 2. Materials and Methods

The present study is a literature review performed on the databases Pubmed, Embase and the Virtual Health Library. The initial search was conducted in January 2023 using the following search terms: “cerebrotendinous xanthomatosis”; “profile”; “cholesterol”; “lipid”; “metabolism” and “metabolic”. All articles initially found in the search were considered for inclusion. We also screened the articles’ reference lists for possible relevant publications.

The records identified were then screened and selected by two independent authors, excluding the articles that did not assess the biochemical or lipid profiles in patients with cerebrotendinous xanthomatosis.

The primary search in Pubmed, Embase and the Virtual Health Library yielded 39, 22 and 20 articles, respectively. After duplicate removal and exclusion of articles not considered relevant for the description of the CTX lipid profile, 67 publications were initially included and had their reference lists analyzed for additional data review. In this later revision, 62 more articles were added, achieving a total of 129 publications reviewed.

## 3. Results

### 3.1. Cholesterol Metabolism

Cholesterol is the precursor of all primary bile acids in mammals, with the human liver producing up to 0.5 g of bile acids per day via two main biosynthetic pathways [1,9,16]. The classic pathway accounts for 90% of total bile acid generation and is initiated by cholesterol 7α-hydroxylase (CYP7A1), an enzyme located in the endoplasmic reticulum of the liver. CYP7A1 catalyzes the 7α-hydroxylation of cholesterol to form 7α-hydroxycholesterol, which is then converted to 7α-hydroxy-4-cholesten-3-one, the final intermediate common to both cholic acid (CA) and chenodeoxycholic acid (CDCA), the two primary bile acids produced [1,2]. Sterol 12α-hydroxylase (CYP8B1) then converts 7α-hydroxy-4-cholesten-3-one to 7α,12α-dihydroxy-4-cholesten-3-one, initiating a route that results in cholic acid. Without this CYP8B1-mediated step, the pathway leads to the production of chenodeoxycholic acid [16,17].

**Figure 1:**
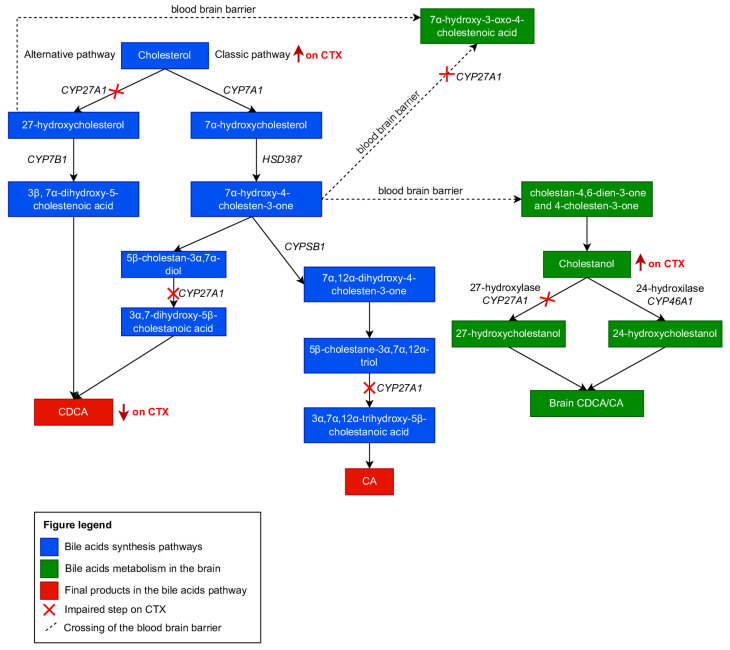
Cholesterol metabolism and changes in cholesterol metabolism present in patients with CTX. Adapted and modified with permission from [3].

The alternative bile acid synthesis pathway is initiated by cholesterol 27-hydroxylase (CYP27A1), a mitochondrial enzyme with ubiquitous distribution throughout body tissues, especially in macrophages. Sterol 27-hydroxylase is involved in the oxidation of different sterol intermediates and is thought to be an important mediator of the removal of cholesterol and atherogenic oxysterols from extrahepatic tissues [9,18]. The first step of the alternative pathway, catalyzed by CYP27A1, is the conversion of cholesterol to 27-hydroxycholesterol, which is then used as a substrate by oxysterol 7α-hydroxylase (CYP7B1) to form 3β, 7α-dihydroxy-5-cholestenoic acid. This intermediate, as well as other oxysterols produced by 25-hydroxylase- or 24-hydroxylase-related pathways, can be converted to chenodeoxycholic acid if transported to the liver [9,17]. These pathways are summarized in Figure 1.

In the brain, the 7α-hydroxy-4-cholesten-3-one produced in the liver can cross the blood–brain barrier and be converted to cholestanol, the 5α-saturated analog of cholesterol which is mainly formed from locally synthesized cholesterol [17,19]. Both cholesterol and cholestanol are oxidized by 27-hydroxylase and 24-hydroxylase (*CYP46A1*), with the latter being a CNS-specific sterol hydroxylase crucial to cholesterol turnover in the brain. The oxysterols produced can cross the blood–brain barrier to systemic circulation and be integrated into bile acid synthesis in the liver [9,17,20].

Under normal circumstances, the 7α-hydroxylation of cholesterol is the rate-controlling reaction for bile acid synthesis, since the activity of *CYP7A1* can be regulated by multiple transcriptional mechanisms [2,10,17,21]. Newly formed bile acids are secreted into the lumen of the small intestine, and their influx back to the liver via enterohepatic circulation constitutes an important negative feedback mechanism as they inhibit *CYP7A1* activity. Cholesterol availability as a substrate is another determinant factor, establishing a link between cholesterol’s de novo synthesis and bile acid production. 

Cholesterol de novo synthesis follows the mevalonate pathway. This process begins with the association of 2 acetyl-coenzyme A molecules, which are catalyzed by the thiolase enzyme, producing acetoacetyl-CoA. Meanwhile, 3-hydroxy-3-methyl-glutaryl-CoA (HMG-CoA) synthase adds another acetyl-CoA-producing HMG-CoA, which is then converted to mevalonate by HMG-CoA reductase [22]. This step plays a critical role in cholesterol synthesis by being a regulated enzyme step in the process and the target of feedback inhibition for the serum cholesterol levels [22,23]. Subsequently, mevalonate undergoes multiple biochemical steps leading to lanosterol, which is formed in another rate-limiting reaction, and finally to cholesterol [22,24].

Another source of cholesterol is one’s diet. Cholesterol absorption occurs mainly in the duodenum and proximal jejunum [25,26] in a process that requires its micellar solubilization [27], as well as cholesterol esterification and intestinal chylomicron assembly, which are carried out by acetyl-CoA acetyltransferase (ACAT2) and microsomal triglyceride transfer protein (MTTP), respectively [28]. 

The Niemann–Pick C1-like 1 (NPC1L1) transporter is responsible for the uptake of cholesterol and plant sterols, such as phytosterol [29]. The other two transporters, adenosine triphosphate protein binding cassette transporters G5 and G8, are co-localized with NPC1L1 in the proximal small intestine. Their function is to stimulate the efflux of unesterified cholesterol from the intestine cells back into the lumen [30]. The remaining cholesterol inside the enterocytes is translocated to the endoplasmic reticulum to be esterified by ACAT2 and later incorporated into chylomicrons by MTTP [31].

The stimulation to produce bile acids reduces the cholesterol levels in the liver, which results in cholesterol de novo synthesis stimulation to provide a substrate for CYP7A1 [17]. However, increased dietary cholesterol intake suppresses de novo synthesis despite biliary acid production [32].

### 3.2. Cholesterol Metabolism in CTX

CTX originates from a deficiency in the activity of the sterol enzyme 27-hydroxylase. This disorder, therefore, compromises the alternative pathway’s function, altering the metabolism of cholesterol-derived sterols [6].

The alternative pathway has CDCA as its final product. With the impairment of this metabolic route already in the initial stages, there is a decrease in the production of this substance [2]. CDCA plays an important role in the metabolism of cholesterol, as it exercises control over the classical pathway through negative feedback, regulating the activity of cholesterol 7α-hydroxylase, which is responsible for the limiting step in the process of bile acid synthesis. Therefore, with CDCA deficiency, there is a stimulation of the classical pathway, leading to the accumulation of intermediates in the plasma and in various tissues (especially cholestanol), in addition to an increase in the excretion of bile alcohols [2,5,8].

Salen and Grundy (1973) used a radioactive cholestanol labeling method in hospitalized patients to observe the metabolism of cholesterol, cholestanol and bile acids in people affected by CTX. They observed that the plasma cholestanol concentration, miscible molecules and cholestanol synthesis rates were 2–5 times higher in CTX patients when compared with the controls [33]. 

Accumulation of lipid metabolites can lead to pathological repercussions throughout the organism [15]. Salen et al. (1991) [16] verified that the proportions of plasmatic cholestanol, which are normally 0.1–0.2% of total cholesterols, were 10–100 times higher in patients with CTX and could reach 2% levels. Furthermore, this proportion can reach up to 50% in the CNS and up to 10% in tendons and the bile.

The loss of function of the enzyme CYP27A1 has been reported in certain types of cancer, such as bladder urothelial carcinoma, breast invasive ductal carcinoma, renal clear cell carcinoma, prostate adenocarcinoma and cutaneous melanoma [34]. Cholesterol availability promotes bladder cancer cell proliferation in vitro, and CYP27A1 activity leads to 27-hydroxycholesterol production and decreased cholesterol levels, thus inhibiting cancer cell proliferation [35]. Thus, CYP27A1 functions as a cellular cholesterol sensor in cancer cells. The relationship between CTX and cancer predisposition as well as a possible effect of CDCA in regulating this pathway in cancer warrant further studies.

The mechanisms by which the accumulation of cholesterol metabolites in the CNS occurs are still not precisely known, as cholestanol has a limited ability to cross the blood–brain barrier (BBB). Among the main hypotheses are that the precursor 7α-hydroxy-4-cholesten-3-one crosses the BBB more easily and can be converted into cholestanol by neurons, microglia and astrocytes. In addition, high levels of cholestanol and apolipoprotein B were found in the cerebrospinal fluid (CSF) of patients with CTX, indicating a possible blood–brain barrier dysfunction in these patients [36].

**Figure 2:**
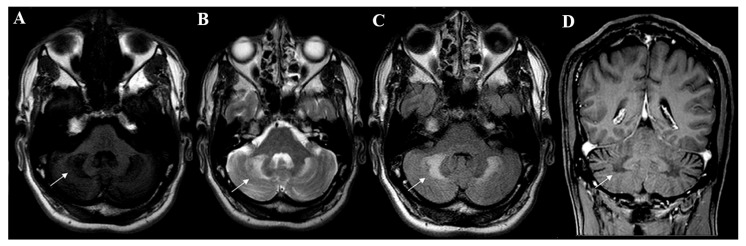
Neuroimaging patterns observed in CTX. Axial brain MRI disclosed signal change in deep cerebellar white matter and dentate nucleus (white arrows) which were hypointense on T1W (**A**) and hyperintense in T2W (**B**) and FLAIR sequences (**C**). Coronal brain MRI showed hypointensity in the deep cerebellar white matter on T1W imaging (**D**).

Inoue et al. (1999) [37] conducted an experiment with rats on a hypercholestanolemic diet and concluded that cholestanol induces apoptosis of the cerebellar neuronal cells, especially for Purkinje cells, a factor that led the rats to present ataxia and tremor. Bogaert et al. (1969) [38]. described the autopsy of ataxic CTX patients, demonstrating the destruction of the dentate and fastigial nucleus, in addition to a reduction in Purkinje cells. Those histologic alterations were manifested in magnetic resonance neuroimaging studies as signal intensity changes in the deep cerebellar white matter and particularly in the dentate nucleus (Figure 2).

### 3.3. Metabolic Abnormalities and Laboratory Diagnosis of CTX

Cerebrotendinous xanthomatosis is a biochemical defect that comes with identifiable metabolic changes. The disruption of the classic pathway of cholesterol metabolism generates precursor accumulation, which is identified in the serum, CSF, urine, bile and traditionally affected CTX tissues. These biochemical markers are useful in both diagnosis and treatment follow-ups.

#### 3.3.1. Serum Profile in CTX

The most evident serum alteration is the increase in cholestanol, which may be 10 times higher than the reference values [1,2,16,39,40]. However, the cholesterol levels are normal or low [39,41,42]. There are sporadic reports of patients with hypercholesterolemia, although this does not seem to be the most frequent presentation [40]. Lower levels of HDL were also detected, along with a decreased cholesterol transportation capacity, which may contribute to atherogenesis and tissue sterol deposits despite normal or low cholesterol levels [16,19,43].

However, increased cholestanol serum levels are not pathognomonic of CTX, as they may be elevated in sitosterolemia, cholestasis or liver diseases. Furthermore, cholestanol levels may be decreased through the use of drugs such as bile acids, statins and steroids [40,44,45]. 

Although the plasmatic cholestanol levels present high accuracy for CTX diagnosis, they do not seem to be relevant for prognosis [46,47]. Nevertheless, patients with the spinal presentation of CTX have lower cholestanol levels when compared with the classic one [48,49].

The disruption of 27-hydroxylase activity may possibly result in undetectable levels for this pathway’s metabolites, particularly 27-hydroxycholesterol and CDCA. The decrease in CDCA negative feedback increases by up to 20 times the pathway controlled by 7α-hydroxylase, leading to accumulation of its products, such as 7α-hydroxycholesterol, 7α-hydroxy-4-cholesten-3-one and 7α,12α-dihydroxy-4-cholesten-3-one, which may serve as auxiliary markers to the follow-up of CTX [1,2,21,39,40,43,50,51]. The levels of 7α-hydroxy-4-cholesten-3-one appear to correlate with the accumulation of cholestanol in the nervous system [52,53,54].

Biliary acids show lower serum levels with an inversion in the physiological CA/CDCA proportion (CDCA predominates in healthy individuals, and CA predominates in CTX) [55]. Conversely, biliary alcohols circulate in excess due to decreased cholesterol side chain cleavage. They present as glucuronides, especially 5β-cholestane-3α, 7α, 12α, 25-tetrol, 5β-cholestane-3α, 7α, 12α, 23R, 25-pentol, 5β-cholestane-3α, 7α, 12α, 24ξ, 25-pentol and 5β-cholestane-3α, 7α, 12α, 22ξ, 25-pentol [16,56]. The excess of biliary alcohols may have a role in blood–brain barrier disruption, increasing its permeability and the accumulation of metabolites in the central nervous system [2,43].

Neonatal cholestasis may be the first manifestation of CTX, presenting conjugated hyperbilirubinemia and elevated transaminases and alkaline phosphatase [6,9,57,58]. These characteristics are also present in other conditions related to inborn bile acid synthesis errors [59].

A possible late manifestation of CTX is osteoporosis. Some reports demonstrate a reduction in 25-hydroxyvitamin D serum levels [60,61,62]. However, Federico (1993) showed normality in the 25-hydroxyvitamin D levels in patients with CTX and osteoporosis, suggesting the possibility of another pathway in this process, which is possibly related to calcium absorption [63]. Koopman et al. (1988) [64] also identified normal levels of vitamin A and decreased levels of vitamin E in patients with CTX. 

The most common changes in serum plasma in patients with CTX are represented in Table 1.

#### 3.3.2. CSF Profile in CTX

Elevated levels of cholestanol and apolipoprotein B and an increased relation between cholestanol and cholesterol have been found in CSF of CTX patients [2,16,39,41]. Höflinger et al. (2021) found an increase of 9–20 times in 25 hydroxylase intermediates, such as 7α, 25-dihydroxycholest-4-en-3-one and 7α, 12α, 25-trihydroxycholest-4-en-3-one, as well as in 7α-hydroxylase pathway metabolites, such as 7α-hydroxy-4-cholesten-3-one and 7α, 12α-dihydroxy-4-cholesten-3-one [1].

#### 3.3.3. Bile, Urine and Tissue Abnormalities in CTX

**Figure 3:**
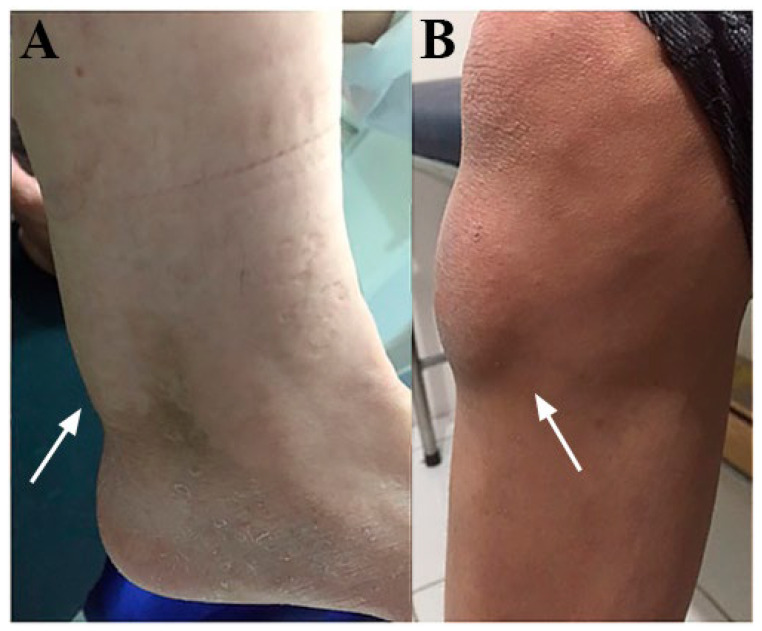
Clinical examination findings in CTX. Note the presence of tendon xanthoma (white arrows) located in the Achilles tendon (**A**) and the anterior tibial tuberosity (**B**). Adapted and modified with permission from [3].

Increased concentrations of cholesterol and cholestanol were registered in tissues and organs, especially in brain white matter and the tendons (Figure 3) [1,36,64,65]. There was also an increase in the cholestanol proportion in several regions of the central nervous system, such as the cerebellum, frontal lobe and parietal lobe [36,64,65]. Postmortem tissues analysis found 10–100 times more cholestanol and 30% more cholesterol when compared with normal tissues. Cholestanol represented up to 2% of sterols in non-xanthomatous tissues and 10% in tendinous xanthomas. The xanthomas in CTX patients have a similar histology to xanthomas in hypercholesterolemia but with higher levels of cholestanol [64]. In addition, 25-hydroxycholesterol and 7α, 12α-dihydroxycholest-4-en-3-one were detected in CTX patients’ brains while being absent in healthy individuals [1], and 27-hydroxychoesterol was not detected in CTX patients, while 7α-hydroxycholest-4-en-3-one was up to 60 times higher in brain tissue [2].

The urine of CTX patients is marked by the presence of biliary alcohols hydroxylated by 25-hydroxylase in large amounts [39,41,48,56,64,65,66,67,68,69]. The main urinary alcohols are cholestane-pentols, with an emphasis on 5β-cholestane-3α, 7α, 12α, 23 and 25-pentol. [50,54,58,64,68,70,71]. Gas chromatography confirmed the presence of norcolic and 23-hydroxycholic acids in non-treated patients’ urine. After treatment, chenodeoxycholic, ursodeoxycholic and hyocholic acids were detected instead of these acids [71]. 

The CTX patient’s bile had a reduced total amount of biliary acids and abnormal composition. Chenodeoxycholic acid production and its proportion were drastically reduced. Colic acid production was also reduced, but its proportion increased [1,14,39,45,68,70,71,72]. Cholesterol was reduced in the bile, while cholestanol was about 10 times higher, with the presence of cholesterol precursors, such as dihydro-lanosterol, lanosterol, and lathosterol. The plant sterol concentration was elevated as well, with the finding of campesterol and sitosterol. Biliary alcohols can be found in the bile and feces of CTX patients, such as 5β-cholestane-3α, 7α, 12α and 25-tetrol and 5β-cholestane-3α, 7α, 12α, 24ξ and 25-pentol [7,8,45,72]. In both the urine and bile, these alcohols were in the glucuronic-conjugated form, while in the feces, they were non-conjugated [48,56,69,72].

#### 3.3.4. Laboratorial Differentials for CTX

Despite being used as a screening tool, the laboratory findings for CTX may confuse this disease with other medical conditions [51]. These conditions must be considered and excluded from the laboratory diagnosis investigation for CTX [48]. The essential distinction factors between CTX and other pathologies of lipid metabolism are the clinical findings, progression and pattern of involvement. However, some reports present laboratory changes that may distinguish it.

#### 3.3.5. CTX Laboratory Findings and Other Diseases

Sitosterolemia is one of the conditions that also presents an increase in the plasmatic levels of cholestanol and 5α-cholestanol, normal or elevated cholesterol levels, elevated activity for the LDL receptors, xanthomas and atherosclerotic disease [4,73,74]. However, there are no neurological symptoms in sitosterolemia. The frequent occurrence of neurological symptoms in CTX, often at an early age, is the main clinical characteristic to differ between sitosterolemia and CTX [47,49,74]. In “non-neurological” forms of CTX, genetic testing seems to be the most reliable tool for distinction [49].

Familial hypercholesterolemia (FH) is another lipid storage disorder that shares some aspects with sitosterolemia. Early neurological symptoms are, again, the main clinical characteristic to differentiate this condition from CTX. Highly elevated cholesterol serum levels (≥310 mg/dl in adults and ≥230 mg/dl in children) are suggestive of FH, although they do not define the condition [75]. These high cholesterol levels are usually not observed in CTX. Unlike CTX, which usually presents normal or decreased LDL levels, both sitosterolemia and FH may manifest highly elevated levels of this apolipoprotein [74,75].

Smith–Lemli–Opitz syndrome (SLOS) is a rare condition caused by defects in cholesterol biosynthesis that presents a variety of clinical manifestations, such as failure to thrive, intellectual deficiency and structural abnormalities such as syndactyly, microcephaly and retrognathism [76]. Despite clinical differences, this condition may have laboratory findings similar to CTX, including normal or lower cholesterol levels and a plasmatic increase in 7-dehydrocholesterol [4].

Other inborn errors of metabolism related to cholesterol or bile acid biosynthesis, obstructive diseases of the biliary tract and non-specific hepatic diseases are all conditions that may alter markers classically related to CTX (specially cholestanol) and must be part of a differential diagnosis [2,4]. 

Some evidence shows that very low levels of 27-hydroxycholesterol (below the detection limit or <5 ng/mL) with highly elevated levels of 7α-hydroxycholesterol and 7α-hydroxy-4-cholesten-3-one may be a characteristic pattern in CTX, differing from the other mentioned conditions [73]. The biliary alcohols cholestan-3β, 5α, 6β-triol (CT) and 7-ketocholesterol (7-KC) are useful for monitoring treatment and diagnosis verification. Both compounds are elevated in the plasma of non-treated patients. Once CDCA treatment starts, there is an expressive reduction in 7-KC, while the CT remains unchanged. This pattern represents a peculiar and thoroughly delimited presentation of CTX when compared with other lipid disorders [77].

### 3.4. CTX Treatment and Its Effect on Metabolism

Chenodeoxycholic acid (CDCA), an exogen bile acid, is considered the standard treatment for the disease and is the most efficient option for reverting the biochemical abnormalities of CTX and modifying the disease’s course [12,13,15,33,61,78,79,80,81,82]. The main objectives of CDCA therapy are to reduce the synthesis of cholesterol, the plasmatic concentrations of cholestanol and the accumulation of cholestanol [18], providing better outcomes for patients [83].

Many studies evaluated the metabolic effects of CDCA therapy in CTX patients. The main metabolic alteration provoked by the treatment was a reduction in serum cholestanol to normal levels in the majority of patients [1,7,12,14,15,33,40,44,53,54,68,69,70,79,84,85,86,87,88,89,90,91]. A reduction in cholestanol titers in the CSF and blood cell membranes was also reported [1,16,68,79,92]. It is possible that CDCA also lowers the BBB permeability to serum cholestanol, reducing the accumulation of this metabolite in the CNS [78,93]. However, the reduction in cholestanol levels in response to therapy is slow compared with other biomarkers [56,83].

Several studies reported that CDCA therapy also lowers the high bile alcohol titers in the serum, bile and urine [1,7,40,44,56,71,79,83,84,85,86,94,95,96]. The normalization of serum bile alcohol levels and urinary and biliary excretion of these metabolites demonstrates CDCA’s capability to inhibit abnormal bile acid synthesis [56,72,83,94]. The reduction in the serum levels of bile alcohols occurs before the normalization of serum cholestanol.

The bile acid serum and urine titers also decrease with treatment [44,56], and CDCA represents the main bile compound with a parallel reduction in cholic and ursodeoxycholic acid. The titers of both the tetrols (represented by 5β-cholestane-3α, 7α, 12α and 25-tetrol) and pentols (represented by 5β-cholestane-3α, 7α, 12α, 23R and 25-pentol, 5β-cholestane-3α, 7α, 12α, 24ξ and 25-pentol and 5β-cholestane-3α, 7α, 12α, 22ξ and 25-pentol) in the urine and serum were reduced [56].

Different papers have observed different cholesterol responses to treatment with CDCA. In general, the serum cholesterol concentration remains stable with treatment [1,40]. Some studies have observed a reduction in cholesterol synthesis with CDCA, but no considerable changes were observed in the serum concentration of this molecule [44,97,98,99]. Despite this, older trials have evaluated cases of patients who had increased serum cholesterol levels after starting this therapy [56,97]. In addition, CDCA caused a reduction in serum HDL [100] and LDL catabolism [97]. It was also seen to reduce the susceptibility of LDL to oxidation [100], preventing the formation of oxidized LDL, an important component of xanthomas [100,101,102,103,104].

Decreases in 7α-hydroxycholesterol [1,98], 7a-hydroxy-4-cholesten-3-one (7α-HCO/7αC4) [1,7,40,54,83,86,87,89,90,91,99], lathosterol and lanosterol were also reported. The levels of other sterols, such as campesterol and sitosterol, were also lowered during treatment [1,40,44,54,83,89,99].

CDCA also induced a reduction in A1 apolipoprotein, B apolipoprotein and albumin in CSF, indicating the reestablishment of the selective permeability of the BBB [79].

The biochemical effects of CDCA therapy in CTX patients are achieved by inhibiting the classic pathway of cholesterol metabolism via negative feedback over the 7α-hydroxylase enzyme [78,83,94,105], reducing abnormal bile acid synthesis [3,40,83,97,99,106]. With the reduction in bile acid synthesis, the production of intermediate metabolites such as 7α-hydroxy-4-cholesten-3-one [49,54,89,90,99] and cholestanol [12,83,94,97,99] is lowered. Excretion of bile alcohols and cholesterol consumption, which stimulates de novo synthesis in a feedback loop, are also inhibited [40,44,83,107].

Aside from CDCA therapy, several studies reported different responses to alternative treatments. A well-studied therapy is the use of cholic acid, which showed clinical improvement or stability in some patients [105,108]. Some of the results obtained with the administration of cholic acid were a reduction in cholestanol blood and CSF levels [58,105,108], in bile acid synthesis, in urinary excretion of bile alcohol and in the abnormal bile acid tilter in urine [105]. Some efficacy was observed in the inhibition of 7α-hydroxylase enzyme activity [105,108]. In a 2019 study with CA, 53% of patients had reduced cholestanol levels, and none had adverse effects [108]. However, cholic acid is not as effective as CDCA, presenting a much smaller capacity for reversing biochemical abnormalities [109,110,111,112,113]. In general, there are contradictory results concerning the utility of cholic acid in CTX treatment and no consensus on its beneficial effects [13,106,114]. Despite this, cholic acid is often used when patients present side effects of CDCA [105,108,115].

Another possible treatment that has been considered is the use of HMG-CoA inhibitors. Controversial results have been observed regarding the clinical and metabolic response to this treatment, which vary among articles [2,5,11]. Several studies reported beneficial effects of statin use in reducing cholestanol [18,58,85,90,108,116,117,118], cholesterol and other plant sterol levels and improving lipoprotein and cholesterol metabolism [13,84,89,119]. In some studies, clinical improvement was observed with the association of statins with CDCA [81,84]. In a consensus statement, using the Delphi method, the association of statins to CDCA was suggested to improve or stabilize the prognoses of patients [13]. However, in other studies, statins failed to decrease abnormal bile acid production or stabilize symptoms [56,119].

Other previously tested treatments that did not achieve satisfactory results are ursodeoxycholic acid (UDCA), LDL apheresis, cholestyramine and clofibrate [13,33,71,83,84,96,109,110,111,120,121,122,123].

### 3.5. Diet Effects in CTX

Currently, there is limited evidence on the association between cerebrotendinous xanthomatosis and dietary patterns, as very few studies investigated either the pathological or therapeutic potentials of one’s diet.

In mice and rats, cholestanol ingestion can increase the cholestanol concentration in the plasma, liver and cerebellum of these animals compared with a control group with no such diet alteration [124,125]. In another study with CTX animal models fed cholestanol, the rats showed lipid accumulation in the Purkinje cells of the cerebellum, while the mice showed corneal opacifications without the presence of xanthomas [126]. A reduction in the plasmatic concentration of cholesterol was also noted in the rat models [124,125], suggesting there might be competition between cholestanol and cholesterol for intestinal absorption, even though cholestanol is known to be more poorly absorbed than cholesterol [125].

The prevalence of cardiovascular disease (CVD) can reach 10–20% in CTX, leading to coronary artery disease at an early age [4,15,127]. In this context, patients with CTX must be screened for CVD and receive dietary recommendations for atherosclerosis prevention [128].

A clinical trial reported clinical improvement in patients treated with a cholesterol-restricted diet in addition to conventional pharmacologic treatment with CDCA. However, a direct association was not established between dietary changes and the observed outcomes [129].

### 3.6. Conclusions

We reviewed the current literature regarding the biochemical aspects of cerebrotendineous xanthomatosis and described the lipid profile of the disease, the pathophysiology causing that profile, the laboratory diagnostics tools, differential diagnoses for an apparent metabolic profile of CTX and the response of the lipidic abnormalities to treatment.

The CTX lipid profile is characterized by impairment in the alternative pathway of cholesterol metabolism, causing a decrease in the metabolites of this pathway, such as CDCA and 27-hydroxicholesterol, and stimulation of the classic pathway through compensatory mechanisms, increasing metabolites and products of this pathway, particularly cholestanol and bile alcohols.

In blood samples, high cholestanol, 7a-hydroxycholesterol, 7α-hydroxy-4-cholesten-3-one and 7α,12α-dihydroxy-4-cholesten-3-one levels, which are associated with low or low-to-normal serum cholesterol, HDL and CDCA levels, raise suspicion for CTX diagnosis. This pattern of laboratory findings may help discriminate CTX from other lipid metabolism-altering diseases, such as sitosterolemia, FH and Smith–Lemli–Opitz syndrome. However, information regarding the onset of the disease and clinical manifestations persists as the most useful approach for differential diagnoses.

The standard treatment for CTX is the administration of CDCA. This treatment has shown the capability to revert both the classic laboratory abnormalities of the disease and its clinical manifestations.

Other therapeutics hypothesized to have beneficial effects in CTX or act as alternatives to CDCA are cholic acid, HMG-CoA inhibitors, ursodeoxycholic acid (UDCA), LDL apheresis, cholestyramine and clofibrate. To date, there is a lack of strong evidence on the beneficial effects of all these alternatives.

## Figures and Tables

**Table 1:** Serum changes in biochemical markers in patients with CTX.

Biochemical Markers	Serum Changes in CTX	Values Found in CTX (Mean ± Standard Deviation)
Cholestanol	Very high	3.42 ± 1.28 mg/dL ^†^
Cholesterol	Normal or low	187 ± 67 mg/dL ^†^
27-hydroxycholesterol	Very low or undetectable	1 ± 1.2 μg/dL ^†^
7a-hydroxycholesterol	High	368 ± 221 μg/dL ^†^
25-hydroxyvitamin D	Normal or low	-
CDCA	Very low or undetectable	0.698 ± 0.315 μg/mL ^◊^
CA	Normal or high	0.213 ± 0.161 μg/mL ^◊^
CA/CDCA	High	1:10 ^□^

CDCA = chenodeoxycholic acid; CA = cholic acid. Reference values adapted from [16] ^□^, [41] ^†^ and [55] ^◊^.

## Data Availability

No new data were created.

## References

[1] Höflinger P., Hauser S., Yutuc E., Hengel H., Griffiths L., Radelfahr F., Howell O.W., Wang Y., Connor S.L., Duell P.B. (2021). Metabolic profiling in serum, cerebrospinal fluid, and brain of patients with cerebrotendinous xanthomatosis. J. Lipid Res..

[2] Koyama S., Sekijima Y., Ogura M., Hori M., Matsuki K., Miida T., Harada-Shiba M. (2021). Cerebrotendinous Xanthomatosis: Molecular Pathogenesis, Clinical Spectrum, Diagnosis, and Disease-Modifying Treatments. J. Atheroscler. Thromb..

[3] Nóbrega P.R., Bernardes A.M., Ribeiro R.M., Vasconcelos S.C., Araújo D.A.B.S., Gama V.C.d.V. (2022). Cerebrotendinous Xanthomatosis: A practice review of pathophysiology, diagnosis, and treatment. Front. Neurol..

[4] Salen G., Steiner R.D. (2017). Epidemiology, diagnosis, and treatment of cerebrotendinous xanthomatosis (CTX). J. Inherit. Metab. Dis..

[5] Nie S., Chen G., Cao X., Zhang Y. (2014). Cerebrotendinous xanthomatosis: A comprehensive review of pathogenesis, clinical manifestations, diagnosis, and management. Orphanet J. Rare Dis..

[6] Gong J.Y., Setchell K.D.R., Zhao J., Zhang W., Wolfe B., Lu Y., Lackner K., Knisely A.S., Wang N.L., Hao C.Z. (2017). Severe Neonatal Cholestasis in Cerebrotendinous Xanthomatosis: Genetics, Immunostaining, Mass Spectrometry. J. Pediatr. Gastroenterol. Nutr..

[7] Verrips A., Dotti M.T., Mignarri A., Stelten B.M.L., Verma S., Federico A. (2020). The safety and effectiveness of chenodeoxycholic acid treatment in patients with cerebrotendinous xanthomatosis: Two retrospective cohort studies. Neurol. Sci..

[8] Russell D.W. (2003). The enzymes, regulation, and genetics of bile acid synthesis. Annu. Rev. Biochem..

[9] Clayton P.T., Verrips A., Sistermans E., Mann A., Mieli-Vergani G., Wevers R. (2002). Mutations in the sterol 27-hydroxylase gene (CYP27A) cause hepatitis of infancy as well as cerebrotendinous xanthomatosis. J. Inherit. Metab. Dis..

[10] Atallah I., San Millán D., Benoît W., Campos-Xavier B., Superti-Furga A., Tran C. (2021). Spinal Cerebrotendinous Xanthomatosis: A case report and literature review. Mol. Genet. Metab. Rep..

[11] Mignarri A., Gallus G.N., Dotti M.T., Federico A. (2014). A suspicion index for early diagnosis and treatment of cerebrotendinous xanthomatosis. J. Inherit. Metab. Dis..

[12] Berginer V.M., Abeliovich D. (1981). Genetics of cerebrotendinous xanthomatosis (CTX): An autosomal recessive trait with high gene frequency in Sephardim of Moroccan origin. Am. J. Med. Genet..

[13] Stelten B.M.L., Dotti M.T., Verrips A., Elibol B., Falik-Zaccai T.C., Hanman K., Mignarri A., Sithole B., Steiner R.D., Verma S. (2021). Expert opinion on diagnosing, treating and managing patients with cerebrotendinous xanthomatosis (CTX): A modified Delphi study. Orphanet J. Rare Dis..

[14] Amador M.D.M., Masingue M., Debs R., Lamari F., Perlbarg V., Roze E. (2018). Treatment with chenodeoxycholic acid in Cerebrotendinous Xanthomatosis: Clinical, neurophysiological, and quantitative brain structural outcomes. J. Inherit. Metab. Dis..

[15] Duell P.B., Salen G., Eichler F.S., DeBarber A.E., Connor S.L., Casaday L. (2018). Diagnosis, treatment, and clinical outcomes in 43 cases with Cerebrotendinous Xanthomatosis. J. Clin. Lipidol..

[16] Salen G., Shefer S., Berginer V. (1991). Biochemical abnormalities in cerebrotendinous xanthomatosis. Dev. Neurosci..

[17] Chiang J.Y. (2013). Bile acid metabolism and signaling. Compr. Physiol..

[18] Burnett J.R., Moses E.A., Croft K.D., Brown A.J., Grainger K., Vasikaran S.D., Leitersdorf E., Watts G.F. (2001). Clinical and biochemical features, molecular diagnosis and long-term management of a case of cerebrotendinous xanthomatosis. Clin. Chim. Acta.

[19] Shore V., Salen G., Cheng F.W., Forte T., Shefer S., Tint G.S., Lindgren F.T. (1981). Abnormal high density lipoproteins in cerebrotendinous xanthomatosis. J. Clin. Investig..

[20] Mast N., Anderson K.W., Lin J.B., Li Y., Turko I.V., Tatsuoka C. (2017). Cytochrome P450 27A1 deficiency and regional differences in brain sterol metabolism cause preferential cholestanol accumulation in the cerebellum. J. Biol. Chem..

[21] Razi S.M., Gupta A.K., Gupta D.C., Gutch M., Gupta K.K., Usman S.I. (2016). Cerebrotendinous xanthomatosis (a rare lipid storage disorder): A case report. J. Med. Case Rep..

[22] Jones P.J.H., Rideout T., Ross A.C., Caballero B., Cousins J.R., Tucker K.L., Ziegler T.R. (2012). Lipids, sterols, and their metabolites. Modern Nutrition in Health and Disease.

[23] Goldstein J.L., Brown M.S. (1990). Regulation of the mevalonate pathway. Nature.

[24] Gill S., Stevenson J., Kristiana I., Brown A.J. (2011). Cholesterol-dependent degradation of squalene monooxygenase, a control point in cholesterol synthesis beyond HMG-CoA reductase. Cell Metab..

[25] Arnesjo B., Nilsson A., Barrowman J., Borgstrom B. (1969). Intestinal digestion and absorption of cholesterol and lecithin in the human. Intubation studies with a fat-soluble reference substance. Scand. J. Gastroenterol..

[26] Borgstrom B. (1960). Studies on intestinal cholesterol absorption in the human. J. Clin. Investig..

[27] Woollett L.A., Wang Y., Buckley D.D., Yao L., Chin S., Granholm N., Jones P.J., Setchell K.D., Tso P., Heubi J.E. (2006). Micellar solubilization of cholesterol is essential for absorption in humans. Gut.

[28] Wang D.Q. (2007). Regulation of intestinal cholesterol absorption. Annu. Rev. Physiol..

[29] Davis H.R., Altmann S.W. (2009). Niemann-Pick C1 Like 1 (NPC1L1) an intestinal sterol transporter. Biochim. Biophys. Acta.

[30] Berge K.E., Tian H., Graf G.A., Yu L., Grishin N.V., Schultz J., Kwiterovich P., Shan B., Barnes R., Hobbs H.H. (2000). Accumulation of dietary cholesterol in sitosterolemia caused by mutations in adjacent ABC transporters. Science.

[31] Gordon D.A., Jamil H. (2000). Progress towards understanding the role of microsomal triglyceride transfer protein in apolipoprotein-B lipoprotein assembly. Biochim. Biophys. Acta.

[32] Di Ciaula A., Wang D.Q., Bonfrate L., Portincasa P. (2013). Current views on genetics and epigenetics of cholesterol gallstone disease. Cholesterol.

[33] Salen G., Grundy S.M. (1973). The metabolism of cholestanol, cholesterol, and bile acids in cerebrotendinous xanthomatosis. J. Clin. Investig..

[34] Petar Brlek P., Bulić L., Weinberger D.G., Bošnjak J., Pavlović T., Tomić S., Dupan Z.K., Borić I., Primorac D. (2023). Successful Treatment of a Rare Cholesterol Homeostasis Disorder Due to CYP27A1 Gene Mutation with Chenodeoxycholic Acid Therapy. Biomedicines.

[35] Liang Z., Chen Y., Wang L., Li D., Yang X., Ma G., Wang Y., Li Y., Zhao H., Liang Y. (2019). CYP27A1 inhibits bladder cancer cells proliferation by regulating cholesterol homeostasis. Cell Cycle.

[36] Menkes J.H., Schimschock J.R., Swanson P.D. (1968). Cerebrotendinous Xanthomatosis: The Storage of Cholestanol Within the Nervous System. Arch. Neurol..

[37] Inoue K., Kubota S., Seyama Y. (1999). Cholestanol induces apoptosis of cerebellar neuronal cells. Biochem. Biophys. Res. Commun..

[38] Van Bogaert L., Philippart M., De Barsy T. (1969). Nouvelles recherches sur la xanthomatose cérébro-tendineuse [New research on cerebro-tendinal xanthomatosis]. Rev. Neurol..

[39] Federico A., Gallus G.N., Adam M.P., Mirzaa G.M., Pagon R.A. Cerebrotendinous Xanthomatosis. GeneReviews®.

[40] Mignarri A., Magni A., Del Puppo M., Gallus G.N., Björkhem I., Federico A., Dotti M.T. (2016). Evaluation of cholesterol metabolism in cerebrotendinous xanthomatosis. J. Inherit. Metab. Dis..

[41] Kuriyama M., Fujiyama J., Yoshidome H., Takenaga S., Matsumuro K., Kasama T., Fukuda K., Kuramoto T., Hoshita T., Seyama Y. (1991). Cerebrotendinous xanthomatosis: Clinical and biochemical evaluation of eight patients and review of the literature. J. Neurol. Sci..

[42] Patni N., Wilson D.P., Feingold K.R., Anawalt B., Blackman M.R., Boyce A., Chrousos G., Corpas E., de Herder W.W., Dhatariya K., Dungan K., Hofland J. (2000). Cerebrotendinous Xanthomatosis. Endotext [Internet].

[43] Björkhem I., Hansson M. (2010). Cerebrotendinous xanthomatosis: An inborn error in bile acid synthesis with defined mutations but still a challenge. Biochem. Biophys. Res. Commun..

[44] De Sain-van der Velden M.G., Verrips A., Prinsen B.H., de Barse M., Berger R., Visser G. (2008). Elevated cholesterol precursors other than cholestanol can also be a hallmark for CTX. J. Inherit. Metab. Dis..

[45] Gelzo M., Di Taranto M.D., Sica C., Boscia A., Papagni F., Fortunato G., Corso G., Russo A.D. (2019). Age-related changes of cholestanol and lathosterol plasma concentrations: An explorative study. Lipids Health Dis..

[46] Pilo de la Fuente B., Ruiz I., Lopez de Munain A., Jimenez-Escrig A. (2008). Cerebrotendinous xanthomatosis: Neuropathological findings. J. Neurol..

[47] Pilo-de-la-Fuente B., Jimenez-Escrig A., Lorenzo J.R., Pardo J., Arias M., Ares-Luque A., Duarte J., Muñiz-Pérez S., Sobrido M.J. (2011). Cerebrotendinous xanthomatosis in Spain: Clinical, prognostic, and genetic survey. Eur. J. Neurol..

[48] Shimazu K., Kuwabara M., Yoshii M., Kihira K., Takeuchi H., Nakano I., Ozawa S., Onuki M., Hatta Y., Hoshita T. (1986). Bile alcohol profiles in bile, urine, and feces of a patient with cerebrotendinous xanthomatosis. J. Biochem..

[49] Sekijima Y., Koyama S., Yoshinaga T., Koinuma M., Inaba Y. (2018). Nationwide survey on cerebrotendinous xanthomatosis in Japan. J. Hum Genet..

[50] Guenzelm A.J., DeBarber A., Raymond K., Dhamija R. (2021). Familial variability of cerebrotendinous xanthomatosis lacking typical biochemical findings. JIMD Rep..

[51] Hong X., Daiker J., Sadilek M., DeBarber A.E., Chiang J., Duan J., Bootsma A.H., Huidekoper H.H., Vaz F.M., Gelb M.H. (2020). Toward newborn screening of cerebrotendinous xanthomatosis: Results of a biomarker research study using 32,000 newborn dried blood spots. Genet. Med..

[52] Pitt J.J. (2007). High-throughput urine screening for Smith-Lemli-Opitz syndrome and cerebrotendinous xanthomatosis using negative electrospray tandem mass spectrometry. Clin. Chim. Acta..

[53] Panzenboeck U., Andersson U., Hansson M., Sattler W., Meaney S., Björkhem I. (2007). On the mechanism of cerebral accumulation of cholestanol in patients with cerebrotendinous xanthomatosis. J. Lipid Res..

[54] DeBarber A.E., Connor W.E., Pappu A.S., Merkens L.S., Steiner R.D. (2010). ESI-MS/MS quantification of 7α-hydroxy-4-cholesten-3-one facilitates rapid, convenient diagnostic testing for cerebrotendinous xanthomatosis. Clin. Chim. Acta..

[55] Beppu T., Seyama Y., Kasama T., Serizawa S., Yamakawa T. (1982). Serum bile acid profiles in cerebrotendinous xanthomatosis. Clin. Chim. Acta..

[56] Salen G., Batta A.K., Tint G.S., Shefer S. (1994). Comparative effects of lovastatin and chenodeoxycholic acid on plasma cholestanol levels and abnormal bile acid metabolism in cerebrotendinous xanthomatosis. Metabolism..

[57] Von Bahr S., Björkhem I., Van’t Hooft F., Alvelius G., Nemeth A., Sjövall J., Fischler B. (2005). Mutation in the sterol 27-hydroxylase gene associated with fatal cholestasis in infancy. J. Pediatr. Gastroenterol. Nutr..

[58] Pierre G., Setchell K., Blyth J., Preece M.A., Chakrapani A., McKiernan P. (2008). Prospective treatment of cerebrotendinous xanthomatosis with cholic acid therapy. J. Inherit. Metab. Dis..

[59] Sundaram S.S., Bove K.E., Lovell M.A., Sokol R.J. (2008). Mechanisms of disease: Inborn errors of bile acid synthesis. Nat. Clin. Pract. Gastroenterol. Hepatol.

[60] Berginer V.M., Shany S., Alkalay D., Berginer J., Dekel S., Salen G., Tint G.S., Gazit D. (1993). Osteoporosis and increased bone fractures in cerebrotendinous xanthomatosis. Metabolism.

[61] Martini G., Mignarri A., Ruvio M., Valenti R., Franci B., Del Puppo M., Federico A., Nuti R., Dotti M.T. (2013). Long-term bone density evaluation in cerebrotendinous xanthomatosis: Evidence of improvement after chenodeoxycholic acid treatment. Calcif. Tissue Int..

[62] Zübarioğlu T., Bilen İ.P., Kıykım E., Doğan B.B., Enver E.Ö., Cansever M.Ş., Zeybek A.Ç.A. (2019). Evaluation of the effect of chenodeoxycholic acid treatment on skeletal system findings in patients with cerebrotendinous xanthomatosis. Turk. Pediatri. Ars..

[63] Federico A., Dotti M.T., Loré F., Nuti R. (1993). Cerebrotendinous xanthomatosis: Pathophysiological study on bone metabolism. J. Neurol. Sci..

[64] Koopman B.J., Wolthers B.G., van der Molen J.C., van der Slik W., Waterreus R.J., van Spreeken A. (1988). Cerebrotendinous xanthomatosis: A review of biochemical findings of the patient population in The Netherlands. J. Inherit. Metab. Dis..

[65] Salen G. (1971). Cholestanol deposition in cerebrotendinous xanthomatosis: A possible mechanism. Ann. Intern. Med..

[66] Bouwes Bavinck J.N., Vermeer B.J., Gevers Leuven J.A., Koopman B.J., Wolthers B.G. (1986). Capillary gas chromatography of urine samples in diagnosing cerebrotendinous xanthomatosis. Arch. Dermatol..

[67] Claesen J.L.A., Koomen E., Schene I.F., Jans J.J.M., Mast N., Pikuleva I.A., van der Ham M., de Sain-van der Velden M.G.M., Fuchs S.A. (2020). Misdiagnosis of CTX due to propofol: The interference of total intravenous propofol anaesthesia with bile acid profiling. J. Inherit. Metab. Dis..

[68] Di Taranto M.D., Gelzo M., Giacobbe C., Gentile M., Marotta G., Savastano S., Dello Russo A., Fortunato G., Corso G. (2016). Cerebrotendinous xanthomatosis, a metabolic disease with different neurological signs: Two case reports. Metab. Brain Dis..

[69] Björkhem I. (2013). Cerebrotendinous xanthomatosis. Curr. Opin. Lipidol..

[70] Haas D., Gan-Schreier H., Langhans C.D., Rohrer T., Engelmann G., Heverin M., Russell D.W., Clayton P.T., Hoffmann G.F., Okun J.G. (2012). Differential diagnosis in patients with suspected bile acid synthesis defects. World J. Gastroenterol..

[71] Wolthers B.G., Volmer M., van der Molen J., Koopman B.J., de Jager A.E., Waterreus R.J. (1983). Diagnosis of cerebrotendinous xanthomatosis (CTX) and effect of chenodeoxycholic acid therapy by analysis of urine using capillary gas chromatography. Clin. Chim. Acta..

[72] Setoguchi T., Salen G., Tint G.S., Mosbach E.H. (1974). A biochemical abnormality in cerebrotendinous xanthomatosis. Impairment of bile acid biosynthesis associated with incomplete degradation of the cholesterol side chain. J. Clin. Investig..

[73] Lütjohann D., Stellaard F., Björkhem I. (2020). Levels of 7alpha-hydroxycholesterol and/or 7alpha-hydroxy-4-cholest-3-one are the optimal biochemical markers for the evaluation of treatment of cerebrotendinous xanthomatosis. J. Neurol..

[74] Moghadasian M.H., Salen G., Frohlich J.J., Scudamore C.H. (2002). Cerebrotendinous Xanthomatosis: A Rare Disease with Diverse Manifestations. Arch. Neurol..

[75] Izar M.C.d.O., Giraldez V.Z.R., Bertolami A., Filho R.D.d.S., Lottenberg A.M., Assad M.H.V., Saraiva J.F.K., Chacra A.P.M., Martinez T.L.R., Bahia L.R. (2021). Atualização da Diretriz Brasileira de Hipercolesterolemia Familiar. Arquivos Brasileiros de Cardiologia.

[76] Schaefer E.J., Tint G.S., Duell P.B., Steiner R.D. (2021). Cerebrotendinous xanthomatosis, sitosterolemia, Smith-Lemli-Opitz syndrome and the seminal contributions of Gerald Salen, MD (1935–2020). J. Clin. Lipidol..

[77] Pajares S., Arias A., García-Villoria J., MacÍas-Vidal J., Ros E., de Las Heras J., Girós M., Coll M.J., Ribes A. (2015). Cholestane-3β,5α,6β-triol: High levels in Niemann-Pick type C, cerebrotendinous xanthomatosis, and lysosomal acid lipase deficiency. J. Lipid Res..

[78] Bonney H., de Silva R., Giunti P., Greenfeld J., Hunt B., Ataxia U.K. (2016). Management of the Ataxias towards Best Clinical Practice. https://www.ataxia.org.uk/wp-content/uploads/2020/11/Ataxia_UK_Medical_Guidelines_Third_Edition._v3m_Dec_2016_-_updated_Sep_2019.pdf.

[79] Salen G., Berginer V., Shore V., Horak I., Horak E., Tint G.S., Shefer S. (1987). Increased concentrations of cholestanol and apolipoprotein B in the cerebrospinal fluid of patients with cerebrotendinous xanthomatosis. Effect of chenodeoxycholic acid. N. Engl. J. Med..

[80] Samenuk P., Koffman B.M. (2001). Chenodeoxycholic treatment of cerebrotendinous xanthomatosis. Neurology.

[81] Mignarri A., Rossi S., Ballerini M., Gallus G.N., Del Puppo M., Galluzzi P., Federico A., Dotti M.T. (2011). Clinical relevance and neurophysiological correlates of spasticity in cerebrotendinous xanthomatosis. J. Neurol..

[82] Ginanneschi F., Mignarri A., Mondelli M., Gallus G.N., Del Puppo M., Giorgi S., Federico A., Rossi A., Dotti M.T. (2013). Polyneuropathy in cerebrotendinous xanthomatosis and response to treatment with chenodeoxycholic acid. J. Neurol..

[83] Stelten B.M.L., Nijeholt G.J.L.A., Hendriks E., Kluijtmans L.A.J., Wevers R.A., Verrips A. (2022). Long-term MRI findings in patients with Cerebrotendinous Xanthomatosis treated with chenodeoxycholic acid. Neurology.

[84] Verrips A., Wevers R.A., Van Engelen B.G., Keyser A., Wolthers B.G., Barkhof F., Stalenhoef A., De Graaf R., Janssen-Zijlstra F., Van Spreeken A. (1999). Effect of simvastatin in addition to chenodeoxycholic acid in patients with cerebrotendinous xanthomatosis. Metabolism.

[85] Bel S., Garcia-Patos V., Rodriguez L., Selvan A., Diaz P., Wolthers B.G., Castells A. (2001). Cerebrotendinous xanthomatosis. J. Am. Acad. Dermatol..

[86] Stelten B.M.L., Huidekoper H.H., van de Warrenburg B.P.C., Brilstra E.H., Hollak C.E.M., Haak H.R., Kluijtmans L.A.J., Wevers R.A., Verrips A. (2019). Long-term treatment effect in cerebrotendinous xanthomatosis depends on age at treatment start. Neurology.

[87] Van Heijst A.F., Verrips A., Wevers R.A., Cruysberg J.R., Renier W.O., Tolboom J.J. (1998). Treatment and follow-up of children with cerebrotendinous xanthomatosis. Eur. J. Pediatr..

[88] Salen G., Tint G.S., Eliav B., Deering N., Mosbach E.H. (1974). Increased formation of ursodeoxycholic acid in patients treated with chenodeoxycholic acid. J. Clin. Investig..

[89] Kuriyama M., Tokimura Y., Fujiyama J., Utatsu Y., Osame M. (1994). Treatment of cerebrotendinous xanthomatosis: Effects of chenodeoxycholic acid, pravastatin, and combined use. J. Neurol. Sci..

[90] Björkhem I., Skrede S., Buchmann M.S., East C., Grundy S. (1987). Accumulation of 7α-hydroxy-4-cholesten-3-one and cholesta-4, 6-dien-3-one in patients with cerebrotendinous xanthomatosis: Effect of treatment with chenodeoxycholic acid. Hepatology.

[91] Björkhem K., Muri-Boberg E., Leitersdorf, Scriver C.R., Beaudet A.L., Sly W.S., Valle D. (2001). Inborn errors in bile acid biosynthesis and storage of sterols other than cholesterol. The Metabolic Bases of Inherited Diseases.

[92] Salen G., Shefer S., Berginer V.M., Stanbury J.B., Wyngaarden J., Fredrickson D.S. (1983). Familial diseases with storage of sterols other than cholesterol: Cerebrotendinous xanthomatosis and sitosterolemia with xanthomatosis. The Metabolic Basis of Inherited Disease.

[93] Oftebro H., Bjorkhem I., Skrede S., Schreiner A., Pederson J.I. (1980). Cerebrotendinous xanthomatosis: A defect in mitochondrial 26-hydroxylation required for normal biosynthesis of cholic acid. J. Clin. Investig..

[94] Batta A.K., Shefer S., Batta M., Salen G. (1985). Effect of chenodeoxycholic acid on biliary and urinary bile acids and bile alcohols in cercbrotendinous xanthomatosis: Monitoring by high performance liquid chromatography. J. Lipid Res..

[95] Batta A.K., Salen G., Shefer S., Tint G.S., Batta M. (1987). Increased plasma bile alcohol glucuronides in patients with cerebrotendinous xanthomatosis: Effect of chenodeoxycholic acid. J. Lipid Res..

[96] Batta A.K., Salen G., Tint G.S. (2004). Hydrophilic 7β-hydroxy bile acids, lovastatin, and cholestyramine are ineffective in the treatment of Cerebrotendinous Xanthomatosis. Metabolism.

[97] Tint G.S., Ginsberg H., Salen G., Le N., Shefer S. (1989). Chenodeoxycholic acid normalizes elevated lipoprotein secretion and catabolism in cerebrotendinous xanthomatosis. J. Lipid Res..

[98] Blaabjerg M., Marjanovic D. (2013). Cerebrotendinøs xantomatose er en sjælden neurologisk sygdom med en specifik behandling [Cerebrotendinous xanthomatosis is a rare disorder, which requires a specific treatment]. Ugeskr Laeger.

[99] Salen G., Meriwether T.W., Nicolau G. (1975). Chenodeoxycholic acid inhibits increased cholesterol and cholestanol synthesis in patients with cerebrotendinous xanthomatosis. Biochem. Med..

[100] Kinoshita M., Kawamura M., Fujita M., Hirota D., Suda T., Taki M., Kusano J., Takao K., Takenaka H., Kubota S. (2004). Enhanced susceptibility of LDL to oxidative modification in a CTX patient:—role of chenodeoxycholic acid in xanthoma formation. J. Atheroscler. Thromb..

[101] Bjorkhem I., Skrede S., Scriver C.R., Beaudet A.L., Sly W.S., Valle D. (1989). Familial disease with storage of sterols other than cholesterol: Cerebrotendinous xanthomatosis and phytosterolemia. The Metabolic Basis of Inherited Disease.

[102] Goldstein J.L., Ho Y.K., Basu S.K., Brown M.S. (1979). Binding site of macrophages that mediate uptake and degradation of acetylated low density lipoprotein, producing massive cholesterol deposition. Proc. Natl. Acad. Sci. USA.

[103] Sparrow C.P., Parthasarathy S., Steinberg D. (1989). A macrophage receptor that recognizes oxidized LDL but not acetylated LDL. J. Biol. Chem..

[104] Witztum J.L., Steinberg D. (1991). Role of oxidized low density-lipoprotein in atherogenesis. J. Clin. Investig..

[105] Koopman B.J., Wolthers B.G., van der Molen J.C., Waterreus R.J. (1985). Bile acid therapies applied to patients suffering from cerebrotendinous xanthomatosis. Clin. Chim. Acta.

[106] Ellis E., Axelson M., Abrahamsson A., Eggertsen G., Thörne A., Nowak G., Ericzon B.-G., Björkhem I., Einarsson C. (2003). Feedback regulation of bile acid synthesis in primary human hepatocytes: Evidence that CDCA is the strongest inhibitor. Hepatology.

[107] Berginer V.M., Berginer J., Korczyn A.D., Tadmor R. (1994). Magnetic resonance imaging in cerebrotendinous xanthomatosis: A prospective clinical and neuroradiological study. J. Neurol. Sci..

[108] Mandia D., Besson G., Lamari F., Castelnovo G., Curot J., Duval F., Giral P., Lecerf J.M., Roland D., Pierdet H. (2019). Cholic acid as a treatment for cerebrotendinous xanthomatosis in adults. J. Neurol..

[109] Makishima M., Okamoto A.Y., Repa J.J., Tu H., Learned R.M., Luk A., Hull M.V., Lustig K.D., Mangelsdorf D.J., Shan B. (1999). Identification of a nuclear receptor for bile acids. Science.

[110] Parks D.J., Blanchard S.G., Bledsoe R.K., Chandra G., Consler T.G., Kliewer S.A., Stimmel J.B., Willson T.M., Zavacki A.M., Moore D.D. (1999). Bile acids: Natural ligands for an orphan nuclear receptor. Science.

[111] Wang H.B., Chen J., Hollister K., Sowers L.C., Forman B.M. (1999). Endogenous bile acids are ligands for the nuclear receptor FXR/BAR. Mol. Cell.

[112] Einarsson C., Hillebrant C.G., Axelson M. (2001). Effects of treatment with deoxycholic acid and chenodeoxycholic acid on the hepatic synthesis of cholesterol and bile acids in healthy subjects. Hepatology.

[113] Huidekoper H.H., Vaz F.M., Verrips A., Bosch A.M. (2016). Hepatotoxicity due to chenodeoxycholic acid supplementation in an infant with cerebrotendinous xanthomatosis: Implications for treatment. Eur. J. Pediatr..

[114] Medscape. https://emedicine.medscape.com/article/1418820-overview.

[115] Bartholdi D., Zumsteg D., Verrips A., Wevers R.A., Sistermans E., Hess K., Jung H.H. (2004). Spinal phenotype of Cerebrotendinous Xanthomatosis—A pitfall in the diagnosis of multiple sclerosis. J. Neurol..

[116] Lewis B., Mitchell W.D., Marenah C.B., Cortese C., Reynolds E.H., Shakir R. (1983). Cerebrotendinous xanthomatosis: Biochemical response to inhibition of cholesterol synthesis. Br. Med. J..

[117] Nakamura T., Matsuzawa Y., Takemura K., Kubo M., Miki H., Tarui S. (1991). Combined treatment with chenodeoxycholic acid and pravastatin improves plasma cholestanol levels associated with marked regression of tendon xanthomas in cerebrotendinous xanthomatosis. Metabolism.

[118] Black M.M., Gawkrodger D.J., Seymour C.A., Weismann K., Champion R.H., Burton J.L., Burns T., Breathnach S. (1998). Metabolic and nutritional disorders. Rook/Wilkinson/Ebling: Textbook of Dermatology.

[119] Peynet J., Laurent A., De Liege P., Lecoz P., Gambert P., Legrand A., Mikol J., Warnet A. (1991). Cerebrotendinous xanthomatosis: Treatments with simvastatin, lovastatin, and chenodeoxycholic acid in 3 siblings. Neurology.

[120] Koopman B.J., Wolthers B.G., van der Moten J.C., Nagel G.T., Waterreus R.J., Oosterhuis H.J.G.H. (1984). Capillary gas chromatographic determinations of urinary bile acids and bile alcohols in CTX-patients proving the ineffectivity of ursodeoxycholic acid treatment. Clin. Chim. Acta.

[121] Koopman B.J., Wolthers B.G., van der Molen J.C., Nagel G.T., Kruizinga W. (1987). Abnormal urinary bile acids in a patient suffering from cerebrotendinous xanthomatosis during oral administration of ursodeoxycholic acid. Biochim. Biophys. Acta.

[122] Dotti M.T., Lütjohann D., von Bergmann K., Federico A. (2004). Normalisation of serum cholestanol concentration in a patient with Cerebrotendinous Xanthomatosis by combined treatment with chenodeoxycholic acid, simvastatin and LDL apheresis. Neurol. Sci..

[123] Ito S., Kuwabara S., Sakakibara R., Oki T., Arai H., Oda S., Hattori T. (2003). Combined treatment with LDL-apheresis, chenodeoxycholic acid and HMG-CoA. J. Neurol. Sci..

[124] Byun D.S., Kasama T., Shimizu T., Yorifuji H., Seyama Y. (1988). Effect of cholestanol feeding on sterol concentrations in the serum, liver, and cerebellum of mice. J. Biochem..

[125] Shefer S., Hauser S., Salen G., Zaki F.G., Bullock J., Salgado E., Shevitz J. (1984). Comparative effects of cholestanol and cholesterol on hepatic sterol and bile acid metabolism in the rat. J. Clin. Investig..

[126] Yousuke S. (2003). Cholestanol metabolism, molecular pathology, and nutritional implications. J. Med. Food.

[127] Valdivielso P., Calandra S., Duran J.C., Garuti R., Herrera E., Gonzalez P. (2004). Coronary heart disease in a patient with cerebrotendinous xanthomatosis. J. Intern Med..

[128] Malco R., Nestor W., Marcelo M. (2021). Cardiac involvement in movement disorders. Mov. Disord. Clin. Pract..

[129] Zhang S., Li W., Zheng R., Zhao B., Zhang Y., Zhao D., Zhao C., Yan C., Zhao Y. (2020). Cerebrotendinous xanthomatosis with peripheral neuropathy: A clinical and neurophysiological study in Chinese population. Ann. Transl. Med..

